# The up-scaling of ecosystem functions in a heterogeneous world

**DOI:** 10.1038/srep10349

**Published:** 2015-05-20

**Authors:** Andrew M. Lohrer, Simon F. Thrush, Judi E. Hewitt, Casper Kraan

**Affiliations:** 1 National Institute of Water and Atmospheric Research, P.O. Box 11-115, Hamilton, 3251, New Zealand; 2 Institute of Marine Science, University of Auckland, Private Bag 92091, Auckland, 1142, New Zealand; 3 Biometry and Environmental System Analysis, University of Freiburg, Freiburg, 79106, Germany

## Abstract

Earth is in the midst of a biodiversity crisis that is impacting the functioning of ecosystems and the delivery of valued goods and services. However, the implications of large scale species losses are often inferred from small scale ecosystem functioning experiments with little knowledge of how the dominant drivers of functioning shift across scales. Here, by integrating observational and manipulative experimental field data, we reveal scale-dependent influences on primary productivity in shallow marine habitats, thus demonstrating the scalability of complex ecological relationships contributing to coastal marine ecosystem functioning. Positive effects of key consumers (burrowing urchins, *Echinocardium cordatum*) on seafloor net primary productivity (NPP) elucidated by short-term, single-site experiments persisted across multiple sites and years. Additional experimentation illustrated how these effects amplified over time, resulting in greater primary producer biomass sediment chlorophyll *a* content (Chla) in the longer term, depending on climatic context and habitat factors affecting the strengths of mutually reinforcing feedbacks. The remarkable coherence of results from small and large scales is evidence of real-world ecosystem function scalability and ecological self-organisation. This discovery provides greater insights into the range of responses to broad-scale anthropogenic stressors in naturally heterogeneous environmental settings.

Scaling up the effects of local processes to larger extents is central to ecology^1^,^2^,^3^,^4^, and critical for predicting the consequences of broad-scale anthropogenic impacts on ecosystem functions that are most tractable to measure at small scales^5^,^6^,^7^,^8^. However, few empirical studies document how the dominant drivers of functioning shift across scales in heterogeneous environments^9^,^10^,^11^,^12^. Moreover, because environmental heterogeneity increases with scale, extrapolations that do not incorporate heterogeneity are prone to inaccuracy^10^,^12^,^13^,^14^,^15^. In this investigation, we integrated detailed empirical data from observational and experimental studies conducted in a range of environmental contexts to evaluate space-, time-, and habitat-dependent influences on seafloor primary productivity in the shallow coastal zone, using previously identified^16^ positive effects of key consumers (burrowing urchins, *Echinocardium cordatum*) on net primary productivity as a focal starting point.

Primary production is a commonly used metric of ecosystem functioning because it fuels food webs and influences biogeochemical processes^5^,^6^,^7^. We examined seafloor primary productivity, the dominant source of productivity in shallow subtidal (<10 m depth) coastal soft-sediment sites^17^,^18^, as an emergent consequence of intrinsic ecological interactions and external drivers. We began by investigating positive and negative feedbacks involving benthic microalgae, inorganic nutrients, macrofauna, and large burrowing urchins (*Echinocardium cordatum*). *Echinocardium* and other spatangoid urchins are key bioturbators of sediments on coastal continental margins worldwide^19^. The impact of urchin bioturbation on primary production in coastal oceans has been theorized based on local scale experimental results that document the provisioning of inorganic nutrients by urchins and concomitant increases in rates of primary productivity by microphytobenthos^16^. Our objective was to provide meaningful empirical tests of the scalability of these results in complex heterogeneous environments.

We predicted that coherent sets of properties would emerge across scales, consistent with the concept of self-organization in ecological systems, as small fast processes mutually reinforce larger slower ones^15^,^20^,^21^. We tested this prediction by measuring net primary production (NPP) across a range of environmental conditions encompassing real-world spatial and temporal heterogeneity, and linking these measurements to habitat- and climate-related drivers operating on differing space and time scales. NPP was quantified by analyzing photosynthetic oxygen production in benthic incubation chambers deployed in different habitat types on multiple occasions. Environmental factors affecting NPP in the short term were predicted to elicit longer-term changes in microalgal standing stock (sediment chlorophyll *a* content, Chla). Therefore, seafloor light intensity and bottom water temperature were logged throughout the NPP incubations; general weather conditions preceding each Chla sampling event were assessed using 30-day and 7-day cumulative meteorological data; and persistent site-related differences in sediment grain size, organic matter content, macrofaunal communities, and *Echinocardium* densities were quantified in conjunction with each of the NPP and Chla measurements for use as explanatory variables in multiple regression analyses.

## Results

**Figure 1:**
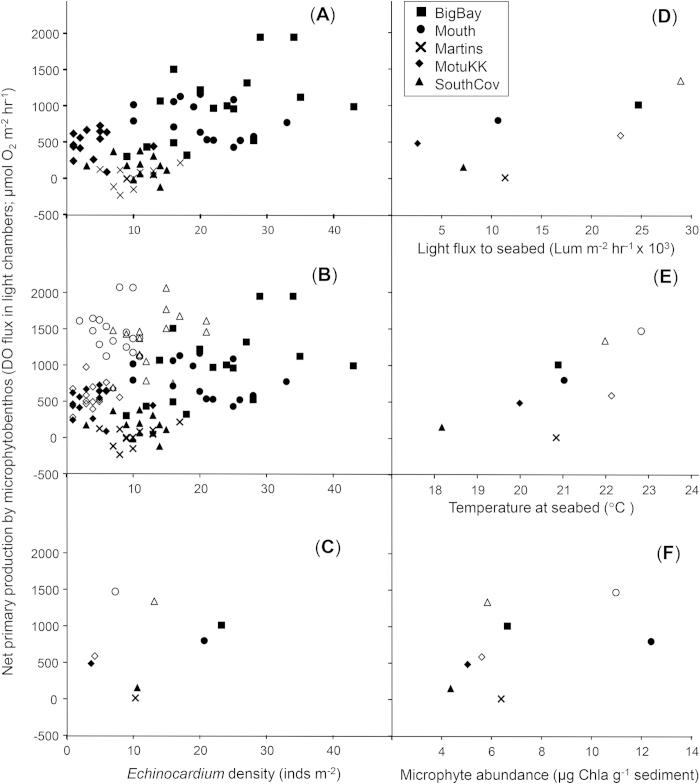
Net Primary Production (NPP) measured in benthic incubation chambers deployed at 5 sites in the Mahurangi Harbour-Kawau Bay study region. (***A***) The general positive relationship between *Echinocardium* density and NPP observed during the first summer of sampling; n = 16 replicates from each of 5 sites sampled between Dec 2006 and Feb 2007. (***B***) *Echinocardium* vs NPP with all of the data from two consecutive summers of sampling included; open symbols are replicates from the second summer of sampling. (***C***) Bivariate scatterplot of site averages. The remaining panels show average NPP at a site versus (***D***) light levels measures at the seabed, (***E***) the temperature of the bottom water, and (***F***) the content of Chla in the sediment.

Surprisingly, given the amount of environmental heterogeneity represented by our sampling sites and times, we observed a significant positive relationship between *Echinocardium* density and NPP across space and time scales (Fig. 1A,B), consistent with results from earlier single site experiments^16^. Significant positive relationships were apparent across sites in two consecutive summers, with five sites sampled the first year, and three sampled again the following year. *Echinocardium* was always the strongest predictor of NPP. Habitat-related variables (*Echinocardium* density, Chla, sediment mud content) explained 63% of the variance in NPP in year one, whereas several weather-related variables contributed significantly to the variance in NPP when considering data from both years-a broader temporal window. The accumulation of rain in the 30 days prior to sampling had a significant negative effect on NPP, whilst the amount of light penetrating to the seabed, and bottom water temperatures at the time of sampling, had significant positive effects on NPP (Fig. 1D,E).

**Figure 2:**
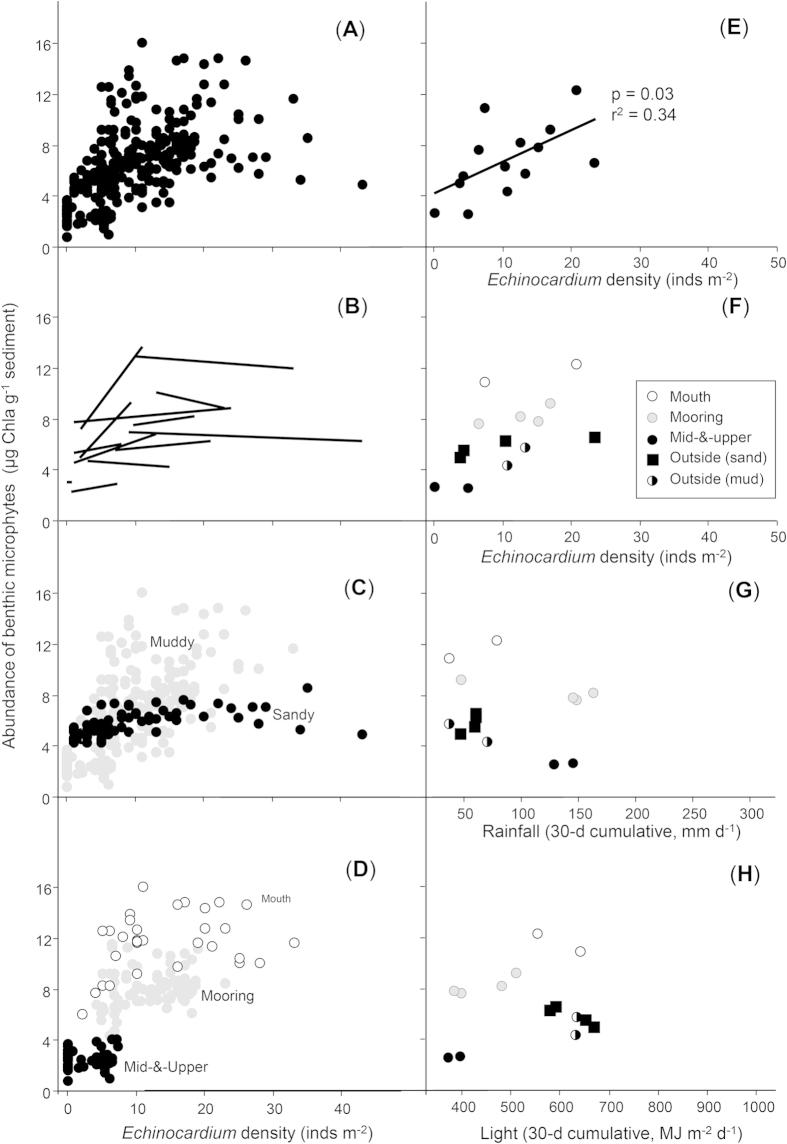
Benthic microalgal standing stock (sediment Chla content) vs *Echinocardium* density in unmanipulated sediments. (***A***) All available data. (***B***) The slopes of relationships during 14 individual sampling occasions from 8 sites. (***C***) Muddy vs sandy sites, above and below 15% average mud content, respectively. (***D***) Three parts of Mahurangi Harbour, with turbidity generally increasing between Mouth and Upper. (***E**-**F***) Plots of averaged *Echinocardium* density and Chla values. (***G**-**H***) The potential influences of environmental factors on sediment Chla content.

*Echinocardium* had consistent effects on NPP and Chla, despite NPP being measured over periods of a few hours and Chla integrating conditions over longer timescales (days to weeks). That is, similar to NPP, we documented a positive relationship between *Echinocardium* density and sediment Chla content in data from 14 sampling occasions among 8 sites (Fig. 2A). The positive *Echinocardium*-Chla trend was strongest in fine sediments that contained >15% mud (Fig. 2C,D). Muddy sites generally have higher organic matter (OM) and pore water nutrient (PWN) concentrations than sandy sites, and urchins probably have a major impact on the release of nutrients from muddy sediments where, in the absence of bioturbation, PWN fluxes would occur slowly by diffusion^22^. In contrast, sandy sites tend to have lower OM and PWN concentrations, given the greater potential for fast physical advection of nutrients from permeable sands by waves and currents^17^.

The mechanisms underpinning these relationships were further explored with manipulative field experiments. After maintaining *Echinocardium* at specific densities for one year, Chla content was highest in plots with the highest densities of *Echinocardium* (p = 0.006, r^2^ = 0.69; Supplementary Fig. S1). However, a similar long-term experiment performed the next year showed no significant trends (p = 0.277, r^2^ = 0.047; Supplementary Fig. S1), despite greater replication and a rigorous design. The first experiment ended in March 2005 after 30 days of fine weather (8.6 mm total rainfall), whereas the second experiment ended in March 2006 following significant storms (46 mm rainfall in the 7 days prior to sampling). This result suggests that the Chla response was controlled by weather conditions that influenced light availability, which, along with nutrients, is a key requirement of photosynthetic primary producers. Cloudy skies and high suspended sediment concentrations associated with stormy weather diminished light levels at the seabed and appeared to negate the positive effects of *Echinocardium* (i.e., nutrient provisioning) on sediment Chla content. With just a single year of fine versus rainy weather, this interpretation is speculative, however, it is also supported by Chla data from sites of similar depth but with differing levels of turbidity (Fig. 2D); sediment Chla content was higher at sites with clearer waters^11^.

**Figure 3:**
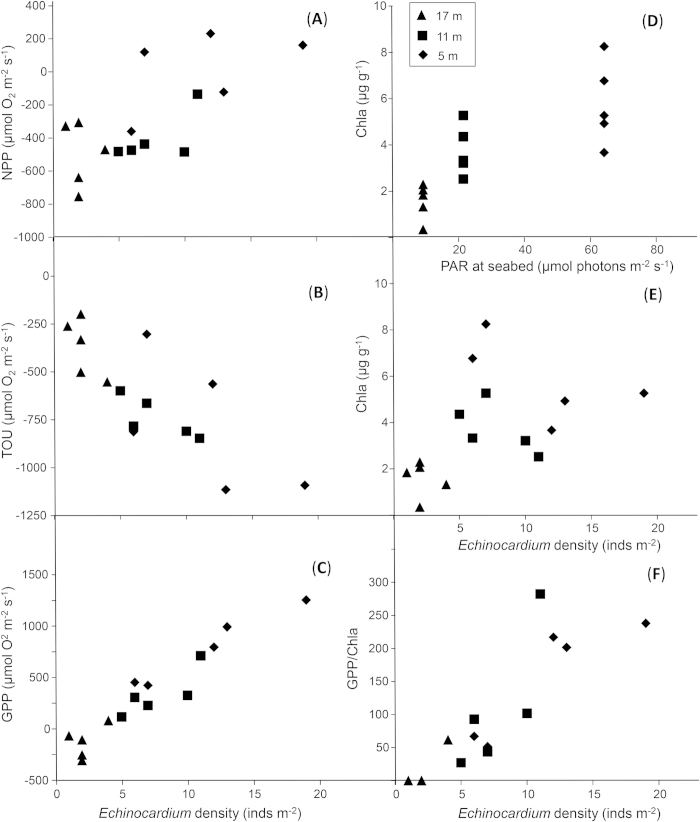
Experimental results from benthic incubation chambers deployed to depths of 17 m (triangles), 11 m (squares) and 5 m (diamonds). (***A***) *Echinocardium* vs NPP, which is dissolved oxygen flux in sunlit benthic chambers. (***B***) *Echinocardium* vs TOU, which is total oxygen utilization measured at night in dark incubation chambers. (***C***) *Echinocardium* vs gross primary production, or GPP, the oxygen produced in the light discounted by the amount of oxygen utilised in the absence of light. (***D***) The relationship between photosynthetically active radiation (PAR) and sediment Chla content; both variables decreased with depth. (***E***) *Echinocardium* density vs Chla. (***F***) *Echinocardium* vs GPP/Chla, which is the rate of gross primary production per unit of Chla. The efficiency of the microphytobenthos in producing oxygen increases with decreasing water depth (or factors correlated with depth).

In a final experiment, we measured fluxes simultaneously at three depths (17, 11 and 5 m), and sediment Chla content and urchin densities were both greatest at the shallowest depth with the most light (Fig. 3). Multiple regression modelling suggested that light was the strongest influence on Chla in this experiment, with *Echinocardium* density and sediment organic matter content also contributing to variation in Chla. Importantly, the density of *Echinocardium* inside the incubation chambers was strongly and positively related to net and gross primary production, and photosynthetic efficiency (Fig. 3).

## Discussion

Positive effects of *Echinocardium* on NPP and Chla seem somewhat paradoxical because *Echinocardium* consumes oxygen and feeds on microalgae. However, feedbacks involving primary nutrient supply to the microalgae explain the paradox^16^. Nutrient enhancement by *Echinocardium* scales with the volume of sediment mixed by populations of these burrowers, whereas grazing rate depends on the (much smaller) volume of sediment passing through urchin guts in a given timeframe^23^. Thus, with increasing urchin density, nutrient provisioning increases faster than grazing rate, producing the emergent pattern through a cross-scale interaction. However, broader external factors controlled seabed interactions as well; light levels at the seabed significantly constrained *Echinocardium*-NPP and *Echinocardium*-Chla relationships during periods of stormy weather and at more turbid sites.

**Figure 4:**
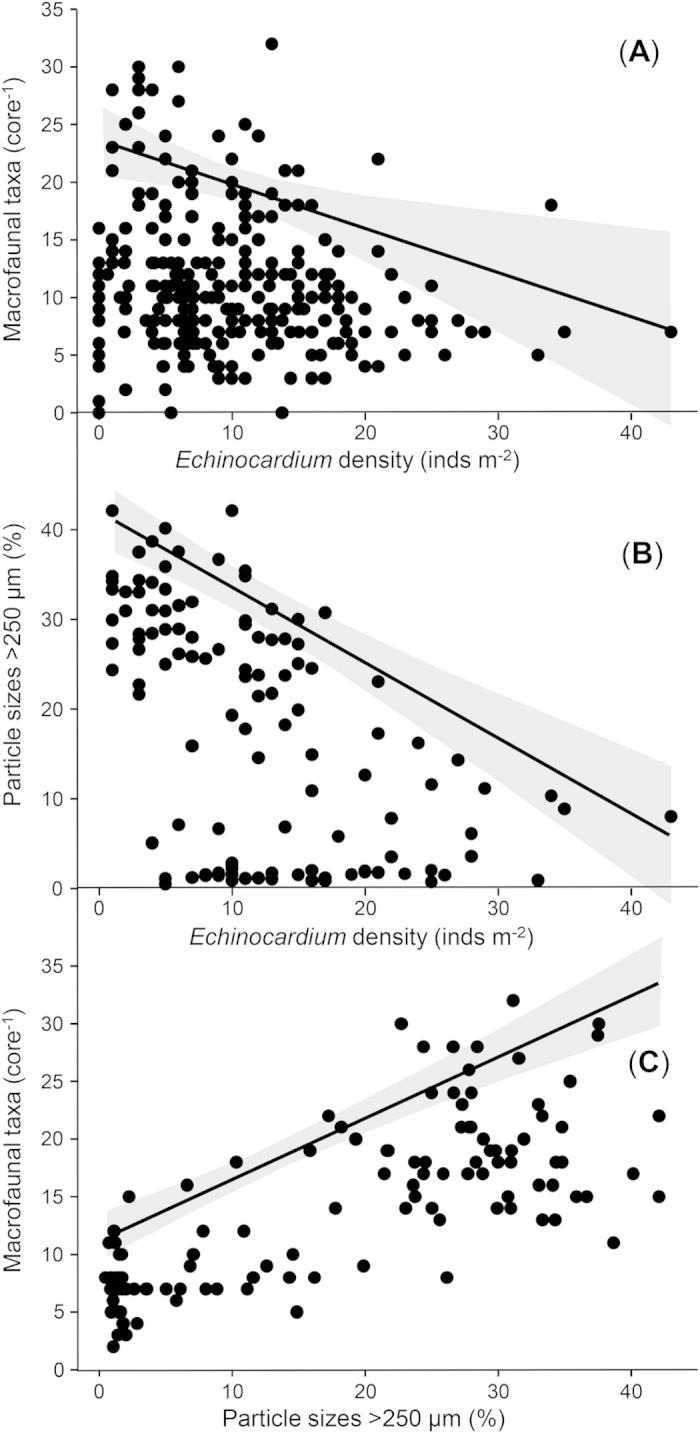
Bivariate plots depicting relationships between *Echinocardium* density, numbers of macrofauna taxa, and sediment granulometry. (***A***) *Echinocardium* vs macrofaunal richness. (***B***) *Echinocardium* vs the percentage of sediment particle sizes >250 μm. (***C***) The percentage of sediment particle sizes >250 μm vs macrofaunal richness. In all panels, significant 90th percentile quantile regression lines (with 95% confidence intervals in grey shading) are drawn.

Mutually reinforcing interactions may also play a role in driving the positive *Echinocardium*-Chla relationship. For example, the relationship appeared to be amplified by negative effects of urchins on other smaller surface-feeding macrofauna that consume Chla^24^ (Fig. 4A). We also documented a negative relationship between *Echinocardium* density and the percentage of particles >250 μm in the sediment (Fig. 4B). Along with fewer urchins in coarse shelly sediments, individual sediment reworking rates may be less, particularly where large bivalve shell fragments interfere with *Echinocardium* burrowing. Altogether, the positive impact of urchins on Chla should be greatest in locations where densities of Chla-consuming macrofauna are low, where urchins can move through the sediment with ease, where concentrations of inorganic nutrients in sedimentary pore waters are elevated, and where, in the absence of bioturbation, nutrients would otherwise diffuse slowly from sediments. Fine muddy sediments lacking coarse shelly material near the mouths of wave-protected harbours appear to meet all of these criteria, and this is where we observed the strongest *Echinocardium*-Chla relationship (Fig. 2C).

The casual observer might regard the positive association between Chla and *Echinocardium* (Fig. 2A) as a simple producer-consumer relationship, with urchins responding to food supply (more urchins in locations with more food). However, our data from multiple habitats and times revealed a much more complicated web of positive and negative interactions, and provided evidence of mutually reinforcing feedbacks and cross-scale interactions. Importantly, the persistence of *Echinocardium*-NPP relationship across steep bio-physical gradients (Fig. 1A,B) instils confidence in the broader generality of the original experimental findings^16^, and suggests that investigations of key ecosystem functions in heterogeneous systems can be up-scaled successfully in some cases.

## Methods

The Mahurangi Harbour-Kawau Bay region of North Island, New Zealand, has a diverse mix of estuarine and open coastal habitats in a relatively small geographical area. To characterise *Echinocardium*-NPP relationships in unmanipulated sediments, fluxes of dissolved oxygen and inorganic nutrients were measured at 5 study sites in this region in the summer of 2007 (Mouth, Martins, Motukete, Big Bay, and South Cove; Supplementary Fig. S2) and again the following summer at three of the sites (Mouth, Motokete, and South Cove) using previously described incubation chamber methodologies^16^. As oxygen is produced by photosynthesis in the presence of sunlight, net primary production (NPP) by microphytobenthos was estimated from mid-day oxygen fluxes in 0.25 m^2^ benthic chambers with transparent lids (n = 16 site^−1^ time^−1^). Fluxes in the dark were used to assess total oxygen respiration and nutrient release without photosynthetic influence. After measuring fluxes, we sampled *Echinocardium*, Chla, macrofauna and sediment characteristics inside each chamber. *Echinocardium*-Chla relationships were developed based on spatially paired data collected in 7 to 10 m water depth at 8 soft-sediment sites on 14 occasions (Supplementary Fig. S2; n ≥ 16 site^−1^ time^−1^). Factors influencing NPP and Chla were determined using multiple regression analyses (see Statistical Analysis section below). The full suite of explanatory variables used to predict NPP included *Echinocardium*, Chla, macrofaunal density, sediment organic matter content, sediment mud %, incident light flux to seabed, ambient water temperature, and cumulative rainfall amount in the 30-d prior to the experiment. Mechanisms driving *Echinocardium*’s relationships to NPP and Chla were further explored in experiments performed in Mahurangi Harbour (see below).

### Sampling Across Substantial Environmental Heterogeneity

Sediment grain size at the study sites ranged from fine sands with up to 30% mud (e.g., inside Mahurangi Harbour) to coarse sands containing large shell fragments (coastal sites with greater wave exposure) (Supplementary Fig. S2). Chla was sampled at all sites using 3 cm diameter, 2 cm depth, sediment cores and processed according to standard methodologies. NPP was assessed using daytime oxygen flux data gathered from benthic incubation chambers, as previously described. All *Echinocardium* >2 cm in length present inside the chambers (0.25 m^2^) were counted. Chla data were always collected in or near chamber bases (0-20 cm away), ensuring tight spatial pairing between *Echinocardium*, Chla, NPP and all other sedimentary variables (including macrofauna, which were collected using 10 cm internal diameter cores and sieved across 500 μm mesh). A range of differing climatic conditions was incorporated by collecting data on 14 occasions between October of 2005 and January of 2008. Meteorological data including rainfall amount (mm d^−1^), sunlight radiation (MJ d^−1^), maximum air temperature (°C), and surface wind run (km d^−1^) were obtained from the nearest weather station (Warkworth), and conditions preceding each sampling event were assessed by calculating 30-d and 7-d cumulative totals (for rain, light and wind) or averages (temperature). In addition, data loggers measuring sunlight intensity and temperature were deployed on the seabed at each site in association with NPP measurements.

### Manipulations of *Echinocardium* Density: Longer-Term Responses of Chla

On 1-March-2004, nine plots were established on the seabed at the mouth of Mahurangi Harbour. Each circular plot (1.6 m diameter) was bordered by a strip of aluminum. The aluminum strips extended ~5 cm above and ~5 cm below the sediment-water interface, serving as a fence that *Echinocardium* could not burrow under or crawl over. No meshed cages/enclosures were used, as these would have altered inputs of sunlight, settling detritus, larval recruits, etc. Our experimental manipulation consisted of collecting urchins by hand and reintroducing them to plots at specific densities. Treatments of 0 inds m^−2^ and 64 inds m^−2^ were established (n = 4 plots each, with one unmanipulated control plot). On 15-March-2005, after a period of one year, final densities of *Echinocardium* were determined from the average of two 0.25 m^2^ quadrats sampled per plot. Sediment Chla content and other sediment characteristics (macrofauna, grainsize, organic matter content, etc.) were then sampled.

Another long-term density manipulation experiment using aluminum-bordered plots was initiated on 12-October-2005. Although the plots in the second experiment were smaller (diameter 1.0 m), replication was greater (28 total plots) and an additional *Echinocardium* density treatment was used (0, 32 and 64 urchins m^−2^). On 14-Mar-2006, after a period of 5 months, final densities of *Echinocardium* were determined by collecting and enumerating all urchins present in each 0.78 m^2^ plot. Chla content and other sediment variables were again collected (one sample per plot) using standard methodologies.

### Effects of ‘Manipulated’ Light Levels

On 1-December-2010, benthic incubation chambers were deployed at three sites of differing depths (17, 11 and 5 m) in Mahurangi Harbour. The three sites were ca. 100 m apart at the mouth of the estuary, and incubations were run simultaneously to ensure standardised temperature and salinity, etc., in all chambers (this was checked with bottom water samples and conductivity-temperature-depth measurements). The three sites were expected to differ primarily according to light levels, due to the attenuation of light with water depth, and measurements of photosynthetically active radiation (PAR) versus depth were made using LiCor sensor casts every 1.5 hrs during daylight hours. Five replicate incubation chambers were deployed to the benthos at each site, without manipulating the sediment or fauna inside the chambers. Measurements of dissolved oxygen flux were made during the day (light), and again at night (dark), to enable us to evaluate net and gross primary production as well as total oxygen utilization rates. Sediment characteristics (Chla, grain size, organic content) and faunal densities (including *Echinocardium*) were then measured inside each chamber.

### Statistical Analysis

Chla and NPP were plotted against *Echinocardium* density and assessed visually. Both individual replicates and site averages were plotted against each other, and data were further divided by site, sediment type or position within Mahurangi Harbour to address particular questions. Combinations of variables working in conjunction to explain NPP and Chla were then determined using multiple regression; explanatory variables were eliminated from initial (full) models by backward selection if not significant at p < 0.15 (proc REG, SAS 9.1). The relative influence of explanatory variables retained in final models significant at α = 0.05 was assessed by examining the significance, magnitude and sign of variable coefficients, based on an analysis of standardized data ranging between 0 and 1. Collinearity diagnostics and variance inflation factors were examined, homogeneity of variance was evaluated by plotting residual vs. predicted values, and normality was assessed via normal probability plots and Shapiro-Wilk tests on residuals to ensure that models met the assumptions of the tests.

## Author Contributions

A.M.L. and S.F.T. designed research; A.M.L. performed research; A.M.L. analyzed the data; A.M.L., S.F.T., J.E.H., and C.K. wrote the paper.

## Additional Information

**How to cite this article**: Lohrer, A. M. et al. The up-scaling of ecosystem functions in a heterogeneous world. *Sci. Rep.* **5**, 10349; doi: 10.1038/srep10349 (2015).

## Supplementary Material

Supporting Information
