# Supplementary material for: The up-scaling of ecosystem functions in a heterogeneous world
Source: Sci Rep. 2015 May 20;5:10349. doi: 10.1038/srep10349 (PMC4438619; doi:10.1038/srep10349)
Supplement: Supporting Information [file srep10349-s1.pdf]

SUPPLEMENTARY INFORMATION

Lohrer A.M., Thrush S.F., Hewit J.E., Kraan, C. The up-scaling of ecosystem functions in a heterogeneous world. Scientific Reports.

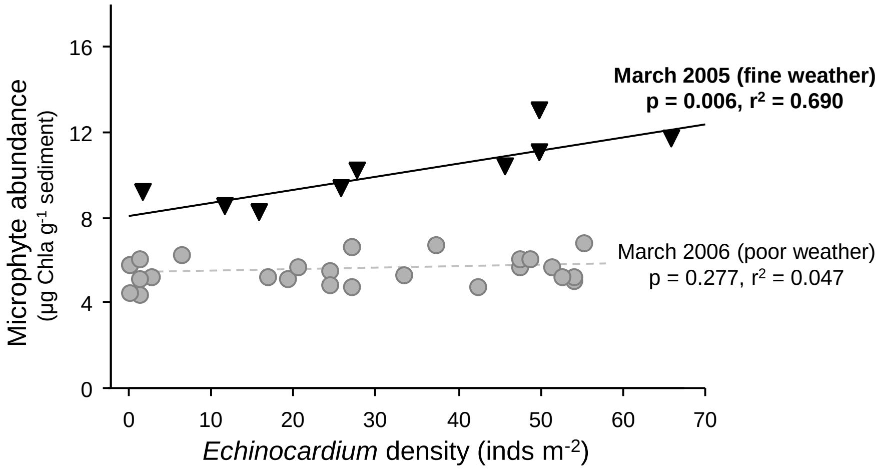

Supplementary Fig. S1. Sediment chlorophyll *a* content (Chla) in plots exposed to varying densities of *Echinocardium* for 1 year (black triangles of Experiment 1, which ended in March 2005) and 5 months (grey circles of Experiment 2, which ended in March 2006). The 2005 results showed a significant positive *Echinocardium*-Chla relationship, whereas the 2006 results were not significant. There were substantial differences in weather conditions (and thus light availability at the seabed) near the end of each experiment that likely modulated experimental results.

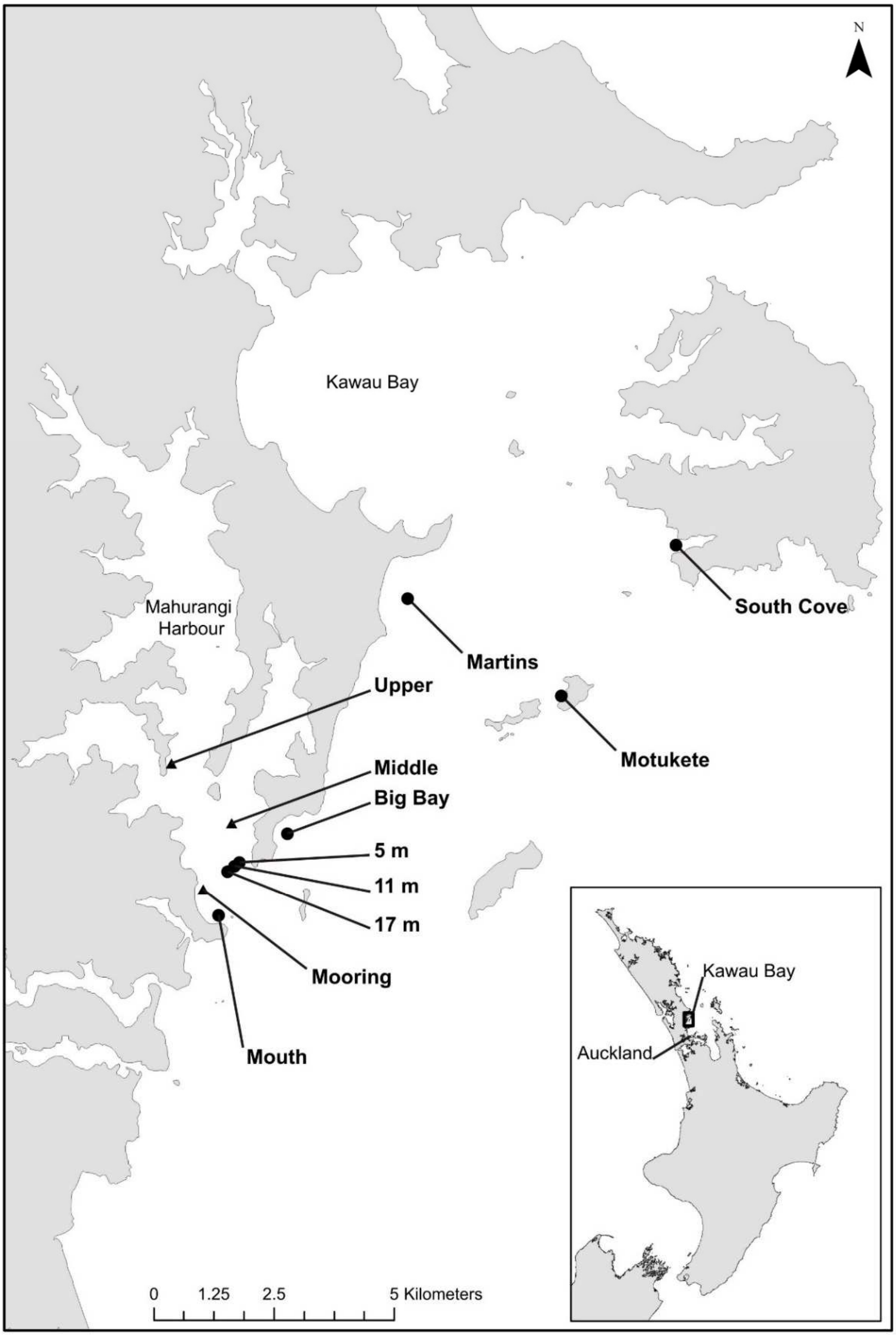

Supplementary Fig. S2. Map (created with ArcMap 10.2.1 software) of study locations in Mahurangi Harbour and Kawau Bay. *Echinocardium* and Chla data were collected at all sites; NPP was measured in benthic incubation chambers at a subset of these sites (marked with filled circles, rather than triangles).
